# Popular Lectures on Anatomy and Surgery

**Published:** 1833

**Authors:** 


### IX. Popular Lectures on Anatomy and Surgery.

We may mention for the information of the students of the West, that Dr. Joseph N. McDowell, of this city, has transferred his Anatomical Museum to Lexington, and will hereafter lecture in that city. Dr. McD. is the nephew, and was the pupil, of the late Dr. Ephraim McDowell, of Danville, Ky. the first surgeon who extirpated a diseased ovary, and for a long time the only lithotomist in the valley of the Mississippi.

As a lecturer on Anatomy, Dr. McD. has, perhaps, no equal of his age in America, being uncommonly clear, energetic and fluent. His course will be a valuable auxiliary to that delivered in the University.

